# Correction: Mabunda et al. Development, Objectives and Operation of Return-of-Service Bursary Schemes as an Investment to Build Health Workforce Capacity in South Africa: A Multi-Methods Study. *Healthcare* 2023, *11*, 2821

**DOI:** 10.3390/healthcare12070723

**Published:** 2024-03-26

**Authors:** Sikhumbuzo A. Mabunda, Andrea Durbach, Wezile W. Chitha, Paidamoyo Bodzo, Blake Angell, Rohina Joshi

**Affiliations:** 1 School of Population Health, University of New South Wales, Sydney, NSW 2052, Australia; 2 The George Institute for Global Health, University of New South Wales, Sydney, NSW 2042, Australia; 3 Australian Human Rights Institute, University of New South Wales, Sydney, NSW 2052, Australia; 4 Health Systems Enablement and Innovation Unit, University of the Witwatersrand, Johannesburg 2000, South Africa; 5 The George Institute for Global Health India, New Delhi 110025, India

## Error in Figure

In the original publication [1], there was a mistake in Figure 3 as published. The original publication had two typos under additional records; first, the ‘Council Minutes and faculty minutes’ were stated to be 609 instead of 270; second, the total ‘Additional records identified through other sources’ were stated as being 867 instead of 418. The corrected **Figure 3** appears below. 

The authors state that the scientific conclusions are unaffected. This correction was approved by the Academic Editor. The original publication has also been updated.

## Figures and Tables

**Figure 3:**
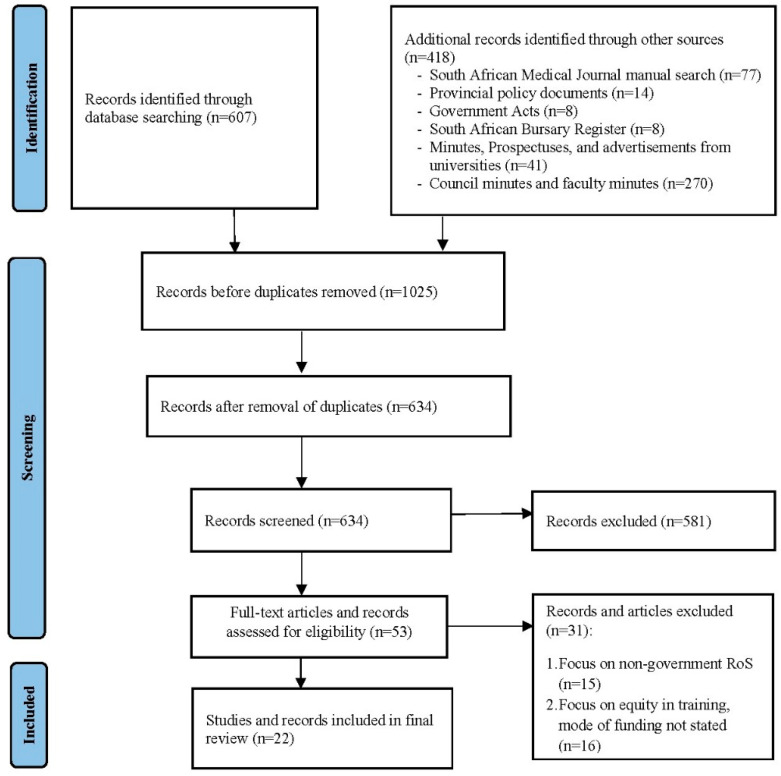
Corrected flow chart—identification of records via electronic databases and manual searches.
