# Supplementary material for: Development, Objectives and Operation of Return-of-Service Bursary Schemes as an Investment to Build Health Workforce Capacity in South Africa: A Multi-Methods Study
Source: Healthcare (Basel). 2023 Oct 25;11(21):2821. doi: 10.3390/healthcare11212821 (PMC10648181; doi:10.3390/healthcare11212821)
Supplement: Supplementary file 1 [file healthcare-11-02821-s001.zip › CARDA_CHECKLIST.pdf]

Checklist for the use and reporting of document analysis (CARDA)

Section and topic	Checklist item	Response/Action
Title	Identify the study involved in document analysis.	Done
Abstract	Identify the methods as mixed (listing the methods) or solely document analysis.	Done
Rationale	Describe the rationale for the use of documents in the study.	Done
Objectives	Provide an explicit statement of the research objective(s) or question(s) of the study.	Done
Eligibility criteria	Specify the eligibility criteria for including documents as data in this specific study.	Done
Document corpus		
Specify the nature of the documents in the corpus:	How many documents there were.	Done
	What kinds of documents were involved (e.g., local curriculum guides and national policy papers	Done
	The documents' media (print, electronic etc.).	Done
	The original purposes of the document where existing documents were used (e.g., Target audience. Who produced them, when and why?)	Done
	Include a table or equivalent documentation of all the documents used (either in the paper or as a supplementary table).	Done
Document provenance	State whether the documents were study-specific or elicited (created as part of data collection, e.g., field notes and diary entries) or existing (extant) documents (e.g., meeting minutes, prospectuses, policy or historical documents).	Done
Where documents were study-specific, specify:	How they were elicited and from whom.	Done
	Whether they were researcher- or participant-created. For example, where diary entries or reflective writing are used and explain why the rationale behind the target group of participants and the guidance given to participants in respect of producing texts.	N/A
	When the documents were created and when they were collected as part of the study (e.g., January 2020 to the end of December 2020).	Done
Where existing documents were used, specify:	How the documents were identified (e.g., archives or websites searched).	Done
	If appropriate, present the full search strategies for document identification, including any filters and limits used (e.g., English language only and particular websites only).	Done
	The data limits of any searches and the rationale for these data limits.	N/A
Document collection and management	How documents were obtained, managed etc.	Done
	Report if any of the documents used are publicly available and where they can be found.	Done

Document quality	Consider the "quality" of the documents and the relation of document quality to the study objectives.	Done
For existing documents:	Were they complete?	Done
	Were there gaps in the documents? Were they redacted?	Done
	Did you need to do more searching or rely on additional documents than planned?	Done
	Were some documents not available or accessible?	Done
For elicited documents:	Did participants engage in the process as intended?	Done
	Were the data comprehensive or sparse?	Done
	How much researcher effort was required to elicit the data, and what might be the implications of researcher interventions (e.g., frequent reminders)?	Done
Reflexivity/positionality (may be placed in the methods or discussion section of your paper)	Role and experience of researchers, experience in DA and positionality.	Done but separated from main document to comply with journal guidelines.
	Consider the potential presence of positionalities, both in a document and of the researcher(s)	Done
Preliminary data analysis	Specify the approach to preliminary or organising data analysis, the methods used to collect data from documents; often using variations on thematic or content analyses.	Done
	Specify if any automation tools were used in the process (e.g., AntConc and Wordsmith).	Done
Document analysis		
Outline the analytical steps taken—what methodology or methods were involved?	Explain whether the analysis focused on content, latent content, linguistics or some other document content or characteristics.	Done
	To what extent and in what ways did analysts immerse or attune themselves to the content, style, subtexts and other dimensions of the documents they analysed?	Done
	Was a theoretical lens used to ensure transferability?	
