# Supplementary material for: Development, Objectives and Operation of Return-of-Service Bursary Schemes as an Investment to Build Health Workforce Capacity in South Africa: A Multi-Methods Study
Source: Healthcare (Basel). 2023 Oct 25;11(21):2821. doi: 10.3390/healthcare11212821 (PMC10648181; doi:10.3390/healthcare11212821)
Supplement: Supplementary file 1 [file healthcare-11-02821-s001.zip › File S1_Data Extraction Tool.pdf]

**Annexure A**

**Data Extraction: Policy Review**

## A. General Information and Eligibility

| | | |
|---|---|---|
| 1. Date form completed | | |
| 2. Name of person extracting data | | |
| 3. Report title | | |
| 4. Publication type | | |
| 5. Type of document | | |
| 6. Publication reference | | |
| 7. Country of publication | | |
| 8. Province | | |
| 9. Policy description | | |
| 10. Decision | Include | |
| | Exclude | |
| 11. Notes (include reasons for exclusion): | | |

*No continuation if excluded.

## B. Context

| **Contextual variable** | **Description** | **Location in document** |
|---|---|---|
| 12. Date of publication | | |
| 13. Version number | | |
| 14. Prior version Review Date | | |
| 15. Current version review Date | | |
| 16. Policy motivation and rationale | | |
| 17. Policy history | | |
| 18. Acts which informed policy | | |
| 19. Target beneficiaries | | |
| 20. Any other contextual issues | | |

## C. Policy Content

| Factor | Description | Location in document |
|---|---|---|
| 21. Classification of beneficiaries | | |
| 22. Beneficiary selection criteria | | |
| 23. Policy objectives and/or purpose | | |
| 24. Skills-mix of beneficiaries | | |
| 25. Conditions of the funding | | |
| 26. Duration of funding | | |
| 27. Budgetary implications | | |
| 28. Administration of scheme | | |
| 29. Beneficiary responsibilities | | |
| 30. Person/body responsible for admitting beneficiaries into scheme. | | |
| 31. Any other factors: | | |

## D. Process Implementation

| Process | Description | Location in document |
|---|---|---|
| 32. Statutory conditions for validity of policy/contract | | |
| 33. Term of policy | | |
| 34. Trigger for review of policy | | |
| 35. Trigger for evaluation of policy | | |
| 36. Skills or service needs determination process | | |
| 37. Beneficiary selection process | | |
| 38. Contract renewal process | | |
| 39. Beneficiary monitoring processes | | |

| | | |
|---|---|---|
| 40. Placement of beneficiaries into services after completion of studies | | |
| 41. Any other processes | | |

## E. Actors Involved

| **Actors** | **Description** | **Location in document** |
|---|---|---|
| | | |
| | | |
| | | |
| | | |
| | | |

## F. Conclusion

| **Remark** | **Description** | **Location in document** |
|---|---|---|
| | | |
| | | |
| | | |
| | | |
| | | |
