# Supplementary material for: Development, Objectives and Operation of Return-of-Service Bursary Schemes as an Investment to Build Health Workforce Capacity in South Africa: A Multi-Methods Study
Source: Healthcare (Basel). 2023 Oct 25;11(21):2821. doi: 10.3390/healthcare11212821 (PMC10648181; doi:10.3390/healthcare11212821)
Supplement: Supplementary file 1 [file healthcare-11-02821-s001.zip › File S3_WC Categorisation of professions.pdf]

Western Cape province's categorisation of funded programmes ***(Western Cape Department of Health 2016)***

Category A: Health Services		Category B: Support services
• Audiology<br>• Clinical Psychology<br>• Clinical Technologist.<br>• Dental Technologist<br>• Dentistry<br>• Dietetics<br>• Emergency Medical Services<br>• Environmental Health<br>• Medical Orthotist and Prosthetics<br>• Medical Physics<br>• Medicine	• Medical Technologist<br>• Nursing<br>• Occupational Therapy<br>• Optometry<br>• Oral Hygienist<br>• Pharmacy<br>• Physiotherapy<br>• Radiography<br>• Social work<br>• Speech Therapy<br>• Speech and language pathology	• Clinical Engineering<br>• Engineering<br>• Financial Administration and/or Management<br>• Health Economics<br>• Human Resources<br>• Industrial Psychology<br>• Industrial Technician<br>• Information Technology (Business Informatics)<br>• Mechatronics<br>• Public Administration<br>• Public Health Administration
