# Supplementary material for: Development, Objectives and Operation of Return-of-Service Bursary Schemes as an Investment to Build Health Workforce Capacity in South Africa: A Multi-Methods Study
Source: Healthcare (Basel). 2023 Oct 25;11(21):2821. doi: 10.3390/healthcare11212821 (PMC10648181; doi:10.3390/healthcare11212821)
Supplement: Supplementary file 1 [file healthcare-11-02821-s001.zip › File S4_MP appointment letter template.pdf]

Mpumalanga Province Internship Appointment Letter Template (2007-2010)

2.5 The termination of your employment is subject to one month's notice by either of the two parties unless both parties agree to a shorter period. The Administration may terminate your service for any reason referred to in sections 16,17 and 19 of the Public Service Act insofar as any reason may apply to you. Termination of employment by the Administration shall occur only after a disciplinary investigation.

2.6 The Administration is not obliged to employ you after completion of your **Internship**, but would like to retain the services of suitable candidates in the Public Sector. You may apply for vacant positions which exist at the time, should you wish to continue your career with the Public Sector. If you had been awarded a bursary for radiography study by the Administration, you must negotiate with the Administration for an appointment in good time.

3. GENERAL POLICY

3.1 You will be stationed at a District/Regional Hospital Complex, but may be rotated and deployed to other hospitals/community health centers/clinics within the district as required according to service needs.

3.2 An annual bonus, equal to 100% of your gross monthly salary, is payable during the month in which your birthday occurs. Should your birthday not coincide with the last month of **Internship**, you will qualify for the payment of a further pro rata annual bonus on the completion of your **Internship.**

3.3 The official hours of work are 40 official hours per week, which extend from midnight Sunday to midnight the following Sunday. In terms of the Public Service Act,1994, the Medical Superintendent/Medical Manager may, at his discretion expect you to work longer hours in the public interest but limited to 80 hours overtime per working month.

3.4 You are expected to complete **24 calendar months of Internship, which will include 22 working days annual leave per each 12 calendar months** . This leave may be granted at any time subject to the recommendation from your supervisor, **but not in the last month of your employment.**

3.5 When deployed to outlying centers as part of the service needs, accommodation will be provided during the period of rotation, dependant during on the commuting distance from the base hospital. A rest room will be provided when rendering after hours service.

3.6 If you are a provincial bursary holder the year of **Intership** will count toward the payment of your bursary obligations.
