# Supplementary material for: Development, Objectives and Operation of Return-of-Service Bursary Schemes as an Investment to Build Health Workforce Capacity in South Africa: A Multi-Methods Study
Source: Healthcare (Basel). 2023 Oct 25;11(21):2821. doi: 10.3390/healthcare11212821 (PMC10648181; doi:10.3390/healthcare11212821)
Supplement: Supplementary file 1 [file healthcare-11-02821-s001.zip › File S5_Beneficiary release letter.pdf]

Correspondence template with beneficiaries who were released (by one province) from obligations in 2020

SUBJECT: RELEASE OF BURSARY HOLDER FROM THE CONTRACTUAL OBLIGATIONS

1. The above matter refers.
2. The Department previously took a decision to develop capacity for it to provide services effectively by providing assistance in fields of study that were scares.
3. To this effect, bursaries were made available to suitably qualifying persons with conditions that were eventually incorporated into the signed bursary agreement, amongst them that, upon completion of the studies, a bursary holder would be obliged to serve the Limpopo Department of Health, for the equal number of years that he or she would have been funded for studies.
4. This meant that all professionals who did Community Service in the Department were absorbed into the service of the Department as a matter of practice, which later included non-bursary holders who would have done Community Service in the Department following demands for fairness in this regard.
5. With the passage of time, sustainability became a challenge, as there was no compatibility between institutional needs and financial capacity.
6. In instances where the number of candidates exceeded the Department's resources, it was resolved that all bursary holders be released from their contracts; and subsequently allow the Department to advertise vacant posts.
7. This memorandum serves to therefore inform you that for the reasons stated above, you are released from your bursary obligations to serve the Department upon completion of your Community Service this year.
8. You will not be indebted to the Department if you find employment elsewhere and you are accorded the same freedom to apply for any post you deem qualified in the Department. Should there be need for clarity, please do not hesitate to contact the Human Resources Management & Development Directorate.

I hope you find the contents of this letter to be in order.

15/07/2020

HEAD OF DEPARTMENT

DATE
