# Supplementary material for: Development, Objectives and Operation of Return-of-Service Bursary Schemes as an Investment to Build Health Workforce Capacity in South Africa: A Multi-Methods Study
Source: Healthcare (Basel). 2023 Oct 25;11(21):2821. doi: 10.3390/healthcare11212821 (PMC10648181; doi:10.3390/healthcare11212821)
Supplement: Supplementary file 1 [file healthcare-11-02821-s001.zip › Table S1-Summary of RSA RoS Policies.pdf]

Table S1. Summary of South African RoS policies for health education

Measures	South African Province					
	**KwaZulu-Natal**	**Limpopo**	**Mpumalanga**	**Northern Cape**	**North West**	**Western Cape**
Earliest draft of signed policy document found	2010	2015	2011	2013	2021	2016
Years of other policy documents found	-2012<br>- A 2021 draft awaiting signatures.	2021	None	2020	None	None
Objectives	-To address the scarcity of skills.<br>-Redress inequalities (racial, gender and people with disabilities).<br>-Poverty alleviation.<br>-Serves as a recruitment and retention strategy by means of service obligation.<br>-Ensure a constant supply of scarce skills for health service delivery.<br>-Assists in the development of career paths for the youth and underdeveloped communities with insufficient disposable income to support their own studies.	-Provide financial assistance to prospective employees.<br>-Recruit and retain scarce, critical and support skills necessary for rendering effective and efficient health services within the province.<br>-Develop and train departmental workforce in order to realise its strategic objectives.<br>-Afford previously disadvantaged groups the opportunity to better their qualifications and be reliably responsive to health	-To develop the Human Resource capacity of both the employees and the citizens of the province.<br>-To maximise the provincial socio-economic capacity and service delivery by improving the supply of required and critical skills.<br>-To address the required, scarce and critical skills deficit in the province and the provincial government, in order to maximise the provincial socio-economic capacity and	-To contribute to the vision and mission of the department.<br>-To enhance service delivery.	-To develop structured needs-based education, training and development opportunities for its current and prospective employees.	-To aid the recruitment and retention of skilled health professionals in areas where it is difficult to recruit and retain suitably qualified candidates.<br>-To align with requirements of current legislation on employment equity and skills development.<br>-To provide selected candidates with sufficient financial aid, subject to the department's affordability, to obtain specialised skills and qualifications through full-time study in a field of Higher Education.<br>-So that the services of beneficiaries may be at the disposal of the department upon completion of their studies, thereby fulfilling

Measures	South African Province					
	**KwaZulu-Natal**	**Limpopo**	**Mpumalanga**	**Northern Cape**	**North West**	**Western Cape**
		and related challenges ravaging communities.<br>-Public administration must be broadly representative of the South African people.<br>-Employment and personnel management practice must be based on ability, objectivity, fairness and need to redress the imbalances of the past to achieve broad representations.<br>-A culture of lifelong learning and the encouragement of employees to develop themselves to assist with career path.<br>-Enhance employee and departmental performance.	service delivery throughput, respectively.<br>-To assist provincial citizens and employees who display potential but lack financial resources to further their studies in fields and priority areas that would add value to the Province.<br>-To assist students who are either citizens of the Mpumalanga Province or employees of the Mpumalanga Government who lack financial resources but are currently furthering or intend furthering their studies in fields and priority areas that would add value to the			the department' s human resource needs.<br>-The bursary scheme will be aligned with the requirement for scarce skills as based on the needs and priorities of the department.

Measures	South African Province					
	KwaZulu-Natal	Limpopo	Mpumalanga	Northern Cape	North West	Western Cape
			Province and the Provincial Government.<br>-To encourage and promote needs-based education.			
Intended beneficiary	-All prospective employee residing within the province.<br>-Must be South African citizens.<br>-They must be pursuing or intending to pursue a health science related qualification.<br>-There is a balance of selected applications, that is, matriculants and those already studying.	- South African Citizen.<br>-Permanent resident of the province (proof of residence must be attached).<br>-From poverty stricken households.<br>-Have passed Grade 12 and satisfied the institution of Higher Learning means test in terms of National Senior Certificate and the National Curriculum Statement respectively.<br>-Permanent employees of the Limpopo Department of Health.	-Citizens of the Mpumalanga Province who come from deep rural communities.<br>-The studies should align with research topics relevant to the needs of the province.<br>-Employment equity plans will be considered.<br>-All districts will be given proportional allocation of bursaries with particular focus on the wards identified for the Comprehensive Rural Development	-Internal bursary recipients should satisfy the minimum required criteria in order to be considered for a bursary allocation.<br>-If for some reason an employee is unable to take up studies within the intended period after bursary allocation, the bursary may be allocated to the next qualifying employee as per the recommendations of the bursary committee presented to the	-	-South African citizens and permanent residents.<br>-Persons who ordinarily reside in the Western Cape province.<br>-Designated groups to be preferred in line with the purpose of this policy.<br>-Bursaries are available from the first year of study.

Measures	South African Province					
	**KwaZulu-Natal**	**Limpopo**	**Mpumalanga**	**Northern Cape**	**North West**	**Western Cape**
		-Selection must take into account, amongst others, gender representation.<br><br>**RSA/Cuban Medical Scholarship**<br><br>-Have passed Grade 12 in the previous years and below the age of 35 years by the end of December of the year of application.<br>-Be in possession of a valid RSA passport.<br>-Satisfy medical tests as prescribed by the Cuban authorities.<br>-Have been historically disadvantaged.<br><br>**MEC special BURSARY Scheme applicant**	Programme (CRDP).<br>-Consideration will be given to issues pertaining to Provincial Succession plans and skills training indicated in the retention strategy for the province.<br>-Internal part-time and full-time students: all employees of the Mpumalanga Provincial Government funded for their development; should register with any higher learning institution in a field that is relevant for the functions of their department; are expected to perform certain functions that require specific educational	Accounting Officer for approval.<br>-The allocation of bursaries shall be subject to availability of funds within a particular financial year.<br>-All bursaries allocated shall be in line with the employee's current post responsibilities and/or in line with the human resource plan of the department.		

Measures	South African Province					
	KwaZulu-Natal	Limpopo	Mpumalanga	Northern Cape	North West	Western Cape
		-Have passed Mathematics, physical Science and English with at least achievement level 6.<br>-Selection must take into account gender representation.<br>- Limited to orphans and destitute applicants who do not have any source of income and who qualify for admission in a particular field or discipline for which the department considers to be very scarce.<br><br>-The Head of Department is not precluded from taking any decision in the interest of the department provided that such a decision is not inconsistent with	qualification level within their department; are not receiving a bursary from National Skills Development Fund during the same period.<br>-External beneficiaries must be needy, unemployed youth residing in the province and potential to pursue a field of study which the province regards as a required scarce and critical skill for its purposes.			

**Measures**	**South African Province**					
	**KwaZulu-Natal**	**Limpopo**	**Mpumalanga**	**Northern Cape**	**North West**	**Western Cape**
		any applicable public service prescripts.				
Service area	-The department reserves the right to deploy the bursary holder where health service delivery is most needed.	-Any location within the Limpopo Provincial Government to address the departmental needs subject to availability of posts.	-Agrees to serve the provincial government or its public entities in any capacity for which the government regards him/her as suitable, for a period of one (1) year for each year's participation in the External Bursary scheme.	Not specified	Not specified	Not specified
Funding model	Settles University invoice.	Settles University invoice.	Settles University invoice.	Fixed amount (capped).	Settles University invoice and assists with transport costs.	Fixed amount (capped).
Cost items*	-Tuition<br>-Stationery<br>-Accommodation<br>- Meals	-Tuition<br>- Prescribed books<br>-Accommodation<br>- Meals<br>- May also consider transport for clinical excursions, medical equipment, stationery, laptops and computer consumables and	-Tuition<br>-Prescribed textbooks only and excluding recommended books<br>-Accommodation<br>- Meals<br>-A fixed R1500.00 monthly stipend	-Fixed amount (internal bursaries)<br>-External: Tuition, examination fees, study materials, transport, stipends (residence fees where applicable)	-Tuition<br>-Stationery<br>-Accommodation<br>- Meals	Fixed amount paid to the University. Beneficiary either finds alternative means to settle the difference or can withdraw the change if there is for their own use.

**Measures**	**South African Province**					
	**KwaZulu-Natal**	**Limpopo**	**Mpumalanga**	**Northern Cape**	**North West**	**Western Cape**
		protective clothing if there is funding.	for students lodging outside institutions' residence and a fixed monthly stipend of R500 for students in residence to cater for travel and toiletry costs.			
Service period	Same as funding duration.	Same as funding duration.	Same as funding duration.	Same as funding duration.	Same as funding duration.	Same as funding duration.
Conditions for breach	-Repeating an academic year.<br>-Non-attendance of classes.<br>-Not serving part or whole contract after completion of studies.	-Repeating an academic year.<br>-Cancellation or termination of studies.<br>-Non-attendance of classes.<br>-Not serving part or whole contract after completion of studies.<br>-Resignation from public service.	-Repeating an academic year.<br>-Non-attendance of classes.<br>-Deviating from the field of study agreed upon without prior written approval.<br>-Expulsion from academic institution.<br>- Failing to produce the examination results as required.<br>-Not serving part or whole contract	-Repeating an academic year.<br>-Non-attendance of classes.<br>-Not serving part or whole contract after completion of studies.	-	-Repeating an academic year.<br>-Non-attendance of classes.<br>-Changing the field of study to another field of study other than that specified in the contract unless authorised by the accounting officer.<br>-Discontinuation of studies.<br>-Not serving part or whole contract after completion of studies.<br>-Resignation or discharge from the public service employment prior to fulfillment of service obligations.

**Measures**	**South African Province**					
	**KwaZulu-Natal**	**Limpopo**	**Mpumalanga**	**Northern Cape**	**North West**	**Western Cape**
			after completion of studies.<br>-Dismissal from employment contract.<br>-Has not (in case of external full-time) submitted an annual declaration on their provincial residency/citizenry status by submitting proof of residency not later than January each year.			-Refusal to accept an offer to work in this department or elsewhere in the public service.
Penalties for breaching	-Repay debt including interest from the date the contract was breached.	-Pay in lump sum the financial assistance offered by the department plus interest as determined from time to time by the National Minister of finance or any responsible organ of state.	-Required to repay all the expenses paid on his/her behalf in the particular year.	-Study debt will be repayable in full or pro-rata depending on the time outstanding before resignation. If the amount payable is insufficient the balance will be recovered from the employee's pension. If the mentioned is insufficient, the official will be	-	-Immediately liable for the repayment of the full outstanding bursary amount.<br>-A bursary will be terminated should the student suspend or terminate his/her studies without prior arrangement with the department.<br>-A student who fails two consecutive years will be held liable for the full repayment of all monies received including money

Measures	South African Province					
	KwaZulu-Natal	Limpopo	Mpumalanga	Northern Cape	North West	Western Cape
				held liable to pay the amount out of own sources before departure from the department.		erroneously paid out by the department.<br>-In calculating the outstanding bursary amount to be repaid by the beneficiary, all periods of full-time or part-time service will be taken into account for the purpose of a pro-rata discount.<br>-Bursary debts must be repaid at the prevailing interest rates as determined by the National Minister of Finance.<br>-In the event that payment has not been made, the matter will be referred to the office of the State Attorney and/or a debt collection agency for collection.
Conditions for Contract variation	-The beneficiary submits a request for extension of the bursary contract due to further studies.<br>-The beneficiary requests a change in subjects, qualification or educational institution.<br>-It becomes apparent that the total period of	-Serve the minimum two (2) years after community service and sign the variation contract with the DoH and serve the department the	-	-As soon as the employee realises that he/she cannot complete his/her studies.<br>-A motivation and recommendation from the HRD sub-directorate must accompany		-The bursary holder's obligations will be considered terminated only if dead, or in appropriate cases of continued ill health, where a written assessment following a comprehensive health examination was made by a qualified medical

Measures	South African Province					
	KwaZulu-Natal	Limpopo	Mpumalanga	Northern Cape	North West	Western Cape
	study will exceed the maximum prescribed duration of the course.	remaining years after completion.		applications for the extension of the duration of the study and approval should be sought from the Accounting Officer.<br>-With regard to the period for which extension may be granted, each case must be considered on merit with a maximum period of 2-years as a guide for extension purposes.<br>-In case of termination of service by a bursar, allowance must be made for the possible existence of other agreements entered into by the bursar, which also gives rise to service obligations.		professional that the student would be unable to fulfill his or her bursary contract and bursary service obligations. Such cases shall be assessed in accordance with the departmental policy on termination of services in respect of ill health which shall require that the student provides the department with medical evidence from a recognised medical specialist. The department shall also have the option to require the student to consult a medical doctor within the department for a second opinion, in appropriate cases and entirely at the department’s discretion.<br>-Deferment may be considered if applied for in writing, for overseas study and research purposes as well as further study in the Republic of South Africa, and which, in the opinion of the Head of the

Measures	South African Province					
	KwaZulu-Natal	Limpopo	Mpumalanga	Northern Cape	North West	Western Cape
				-In case of death or the bursar being incapacitated by any mental or physical disability for the refund of any moneys that may be owed to the department must be communicated to the Accounting Officer to obtain authority to write-off the debt.		Department will be in the interest of the department.<br>-For the purposes of gaining relevant experience locally and abroad in a specific field which, in the opinion of the Accounting Officer will be in the interest of promoting effective service delivery within the department.<br>-Deferment may be granted for a maximum period of two years. Only in exceptional cases will consideration be given to periods that exceed two continuous years.<br>-Students who apply for deferment in order to go overseas for study and/or work purposes must submit a formal letter of Guarantee of payment ("the bank guarantee") to the department from a recognised South African Commercial bank for the full outstanding bursary amount with their application for deferment.

**Measures**	**South African Province**					
	**KwaZulu-Natal**	**Limpopo**	**Mpumalanga**	**Northern Cape**	**North West**	**Western Cape**
						-Should the student fail to return to complete the Bursary Service Obligations then the bank guarantee will be redeemed to cover the full outstanding bursary debt.
Other Countries	Cuba	Cuba	-Cuba<br>-Russia	Cuba	Cuba	None
Acknowledgement of previous versions	Yes, 2010 version acknowledges 19 August 2003 version.	No	No	No	No	No
Review date	-	The review of the policy shall be influenced by the socio-economic challenges, development and national priorities and mandates.	-	After 3-years from the publishing date.	-	-

*Transport is also included for internationally enrolled students;
Except for Western Cape and Gauteng all other provincially funded nursing students at college get a monthly stipend in addition to their study fees being settled by the scheme;
Tuition is inclusive of registration fees;
Eastern Cape, Free State and Gauteng provinces do not have signed policy documents.
