# Supplementary material for: Development, Objectives and Operation of Return-of-Service Bursary Schemes as an Investment to Build Health Workforce Capacity in South Africa: A Multi-Methods Study
Source: Healthcare (Basel). 2023 Oct 25;11(21):2821. doi: 10.3390/healthcare11212821 (PMC10648181; doi:10.3390/healthcare11212821)
Supplement: Supplementary file 1 [file healthcare-11-02821-s001.zip › Table S2-Selected 1965 RSA schemes .pdf]

Table S2. Selected South African RoS schemes advertised in 1965

Scheme type	Funding administration	Academic programmes funded	Years of funding	Funding model	Beneficiary characteristics	Beneficiary obligations
Bursary-loan	The Public Service Commission and the National Department of Education, Arts and Science	• Medical studies	Third to sixth year	• Capped per programme of study and assessed personal circumstance of each beneficiary<br>• Maximum of R400 per annum for third and fourth year.<br>• Maximum of R600 for the fifth and sixth academic year.	• South African citizen<br>• In the opinion of the Commission, free of any mental or physical defect, disease or infirmity which would be likely to interfere with the proper carrying out of their duties as public servants.<br>• In possession of a matriculation or equivalent, with Afrikaans and English as subjects.<br>• Successful completion of part of a degree or course in any of the fields sponsored will be a recommendation.	• Sign a binding contract.<br>• Repay 40% of the loan at 6% interest per annum.<br>• Discharge the balance of 60% of funding support through service to the public health sector depending on the amount of funding received ranging from a minimum of 2-years to a maximum of 7-years.<br>• Holders of bursary loans for courses for which practical training is compulsory for degree purposes, must do such practical work during holiday periods in Government departments and administrations to be determined by the Public Service Commission. During such periods students will normally receive the remuneration paid
		• Dietetics<br>• Physiotherapy<br>• Speech Therapy<br>• Pharmacy<br>• Social work<br>• Microbiology<br>• Psychology	Second to final year	• Full-time study: R200 – R400 per annum (maximum of R300 per annum for women)<br>• Part-time study: maximum R120 per annum.		

						to university students who are employed by the state.<br>• Medical graduates commit to serve their internship in an approved South African hospital after obtaining the MBChB qualification and to obtain the internship within thirteen months of qualification.
Bursary	*Transvaal hospital services bursary	Medical studies	Fourth to sixth year	Maximum of R400 per annum.	• Not stated	• Service a Transvaal provincial hospital from internship, whereafter serve the province for every three hundred-Rands or portion thereof of funding received.
		• Physiotherapy<br>• Occupational therapy<br>• Radiography	•	• Covers all tuition fees.<br>• Makes provision for a training allowance of R40 per month.		• Sign a binding contract to serve a Transvaal Provincial hospital after completion of training for at least one year for each year of free training, to a maximum of three years of service.<br>• In the event of breach of contract:<br>- All tuition and examination fees paid

						out must be refunded in full.<br>- 50% of all monthly allowances paid out during their period of training must be refunded.<br>-Should a beneficiary complete the course but fail to serve the contract in full, then the amount due as "…liquidated damages…" shall be reduced on a pro-rata basis in respect of each calendar month of service rendered after completion of training.
Scholarship	Julius Robinson Charitable and Educational Trust scholarship	• Medical studies	Maximum of six years	• R800 per annum.	• Meritorious, non-European (non-white).<br>• Evidence of qualities of character as well as intellectual ability as shown by the candidate's previous scholastic record	• Signed a contract to practice in South Africa or "…Protectorates…" for a period of at least three years (not specific to public or private sector, urban or rural)
*A similar scheme falls under the Provincial Administration of the Cape of Good Hope.						
